# Predicting risk of impending cognitive decline in asymptomatic individuals with early Alzheimer’s disease: Insights from cortical diffusion MRI

**DOI:** 10.1162/IMAG.a.1037

**Published:** 2025-11-24

**Authors:** Devon K. Overson, Trong-Kha Truong, Jeffrey R. Petrella, David J. Madden, Yixin Ma, Kim G. Johnson, Andy J. Liu, Richard J. O’Brien, Heather E. Whitson, Allen W. Song

**Affiliations:** Brain Imaging and Analysis Center, Duke University, Durham, NC, United States; Medical Physics Graduate Program, Duke University, Durham, NC, United States; Department of Radiology, Duke University Medical Center, Durham, NC, United States; Duke Center for the Study of Aging and Human Development, Duke University, Durham, NC, United States; Department of Psychiatry and Behavioral Sciences, Duke University Medical Center, Durham, NC, United States; Department of Psychology and Neuroscience, Duke University, Durham, NC, United States; Department of Neurology, Duke University, Durham, NC, United States; Department of Medicine, Duke University, Durham, NC, United States

**Keywords:** diffusion MRI, cortical columns, microstructural neurodegeneration, gray matter, Alzheimer’s disease, cognitive decline

## Abstract

Neurodegeneration associated with Alzheimer’s disease (AD) can start at the microstructural level years before cognitive symptoms; yet, it has been difficult to definitively detect these early changes to plan effective treatments. Using a cortical column-based analysis of high-resolution diffusion magnetic resonance imaging data, we aim to identify early microstructural neurodegeneration in the gray matter across different cortical depths and regions. We compared four groups of participants across a spectrum of cognitive decline (n = 60): cognitively normal amyloid-negative (normal controls), cognitively normal amyloid-positive (stage-1 AD), mild cognitive impairment (MCI), and AD dementia. Our results showed progressive increases in radial diffusivity across these groups in cortical regions associated with AD, and our analysis in individual asymptomatic stage-1 AD participants was able to differentiate such diffusivity increases to predict risk of impending cognitive decline in 2 participants who had cognitive decline and MCI diagnosis at their follow-up clinical examination and 11 participants who did not.

## 1 Introduction

Neurodegenerative processes associated with Alzheimer’s disease (AD) can occur years before the onset of cognitive symptoms (Apostolova & Thompson, 2008; Vickers et al., 2016). However, existing biomarkers for AD cannot detect the earliest microstructural neurodegeneration at the presymptomatic stage to reliably predict future clinical symptoms (Weston et al., 2015). For example, volumetric changes, such as cortical atrophy, derived from structural magnetic resonance imaging (MRI), are often detected at later symptomatic stages, when it is too late for the clinical course to be altered by available treatments. On the other hand, abnormal levels of amyloid-β (Aβ) proteins can be found early, but do not definitively predict future cognitive impairment (Jack Jr, Wiste, et al., 2024; Resnick & Sojkova, 2011; Sperling et al., 2014). Thus, there is a need to develop novel biomarkers that can reliably detect early microstructural neurodegeneration and help assess risk of impending cognitive decline before the onset of symptoms. The ability to better differentiate dementia risks at the presymptomatic stage is critical to guide clinical interventions (e.g., early medications) to effectively delay the onset or slow the progression of symptoms (van Dyck et al., 2023).

In this work, we seek to achieve this goal by developing a novel diffusion MRI (dMRI) methodology to investigate early microstructural neurodegeneration in the cortical gray matter. Previous cross-sectional (Weston et al., 2015; Montal et al., 2018; Weston et al., 2020) and longitudinal (Spotorno et al., 2024; Sun et al., 2024) dMRI studies have reported increases in cortical diffusivity along the AD continuum and over time, including at the presymptomatic stage. Increased diffusivity at baseline was also found to predict faster subsequent cognitive decline and clinical progression, with a higher sensitivity than cortical thickness (Gagliardi et al., 2023; Rodriguez-Vieitez et al., 2021). Such increases in cortical diffusivity are thought to reflect synaptic, dendritic, and/or axonal degeneration resulting in a breakdown of microstructural barriers (e.g., cell membranes, intracellular organelles, myelin) and an increase in extracellular water, but the exact mechanisms and relationships with Aß and tau pathologies, glial activity, neuroinflammation, and microvascular pathology are still under investigation (Gagliardi et al., 2023; Montal et al., 2018; Rodriguez-Vieitez et al., 2021; Weston et al., 2015, 2020).

Importantly, neurodegeneration in AD is not uniform across different cortical layers or cortical regions, which have distinct and selectively vulnerable neuronal populations. For example, histological studies found neuronal loss to be more pronounced in layers II and IV of the entorhinal cortex (Astillero-Lopez et al., 2022; Gómez-Isla et al., 1996) and in layers III and V of the parahippocampal gyrus (Thangavel et al., 2008). However, previous dMRI studies (Gagliardi et al., 2023; Montal et al., 2018; Rodriguez-Vieitez et al., 2021; Weston et al., 2015, 2020) based on single-shot echo-planar imaging (EPI) were limited by a low spatial resolution (~2 mm or lower) compared with the average cortical thickness (2.5 mm) across the brain (Fischl & Dale, 2000), resulting in a poor spatial specificity and mostly isotropic diffusivity in gray matter. Additionally, they used voxel-, region-, or surface-based analyses that did not take into account any cortical depth dependence, except for one ex vivo study (Zhao et al., 2025).

Here, we thus employ a novel cortical column-based analysis (Ma et al., 2023) of high-resolution (1 mm) dMRI data acquired with multi-band multi-shot EPI to better resolve the microstructure of cortical gray matter and assess how the diffusivity varies across different cortical depths and regions. Using four groups of participants across a spectrum of cognitive decline, we perform both group and individual participant analyses to evaluate (i) whether the diffusivity progressively increases across these groups in regions associated with AD and (ii) whether these increases in diffusivity can differentiate risk of impending cognitive decline among asymptomatic participants, with the long-term goal to enable individualized risk assessment and potentially early and more effective treatments.

## 2 Methods

### 2.1 Participants

Sixty participants were recruited by the Duke University and University of North Carolina at Chapel Hill Alzheimer’s Disease Research Center. They (or their legal caregivers) provided written informed consent to participate in this study approved by the Duke University Health System Institutional Review Board. These participants included 17 cognitively normal Aß-negative participants (group 1, normal controls), 16 cognitively normal Aß-positive participants (group 2), 15 participants with clinically diagnosed amnestic mild cognitive impairment (MCI) (group 3), and 12 participants with clinically diagnosed AD dementia (group 4). Clinical diagnosis was based on a multidisciplinary clinical consensus panel of neurologists, psychiatrists, neuropsychologists, and research coordinators using the current Alzheimer’s Association framework for diagnosing and staging AD (Jack Jr, Andrews, et al., 2024), which was updated from the 2018 framework based on the A/T/(N) criteria (Jack Jr et al., 2018). In the updated framework, abnormality on specific biomarkers, such as the Aß42/40 ratio, is sufficient to diagnose AD, and the cognitively normal Aß-positive participants from group 2 are classified as stage-1 AD. The participants’ Aβ42/40 ratio was measured on the Fujirebio Lumipulse platform (Fujirebio, Ghent, Belgium) by using cerebrospinal fluid (CSF) acquired through lumbar puncture. Aß-negative and Aß-positive participants were defined as those with an Aß42/40 ratio above or below the previously established cutoff of 0.058, respectively (Esquivel et al., 2021; Willemse et al., 2020).

The four groups were approximately age matched (mean ± standard deviation = 69.1 ± 6.1, 67.1 ± 8.9, 69.9 ± 7.7, and 69.5 ± 9.3 years) and included 82%, 69%, 33%, and 83% of females, respectively. All participants were right handed, except for one participant from group 4 whose handedness was not reported. Depending upon their date of initial enrollment, follow-up clinical examinations were also performed on 71%, 81%, 53%, and 58% of participants from groups 1–4, respectively, by reassessing their cognition and functioning at approximately 1-year intervals to detect any changes.

### 2.2 MRI data acquisition and pre-processing

All MRI data acquisitions were performed on a 3T Ultra-High Performance MRI scanner (GE Healthcare, Milwaukee, WI) equipped with a 48-channel head coil and a 60-cm gradient system with a maximum amplitude of 100 mT/m and a maximum slew rate of 250 T/m/s. For each participant, whole-brain dMRI data were acquired with a spin-echo multi-band, multi-shot EPI sequence (acquisition plane = axial, number of bands = 2, number of shots = 4, repetition time = 8767 ms, echo time = 61 ms, voxel size = 1 mm isotropic, number of b = 0 image volumes = 2, number of diffusion-weighting directions = 25, b-value = 800 s/mm^2^, scan time ≈ 17 min). This scan also included an additional b = 0 image volume acquired with a reverse phase-encoding polarity to correct for susceptibility-induced distortions. Additionally, whole-brain T_1_-weighted anatomical images were acquired with a 3D magnetization-prepared rapid acquisition gradient echo (MPRAGE) sequence (acquisition plane = axial, repetition time = 2077 ms, inversion time = 900 ms, echo time = 3 ms, flip angle = 8^o^, voxel size = 1 mm isotropic), and whole-brain T_2_-weighted anatomical images were acquired with a 3D fluid attenuated inversion recovery (FLAIR) sequence (acquisition plane = sagittal, repetition time = 6300 ms, inversion time = 1813 ms, echo time = 105 ms, voxel size = 1 mm isotropic).

### 2.3 dMRI data reconstruction and preprocessing

The dMRI data were reconstructed with a multi-band multiplexed sensitivity-encoding (MUSE) algorithm (Chen et al., 2013) to correct for shot-to-shot motion-induced phase errors. The resulting images were then preprocessed by applying denoising (Cordero-Grande et al., 2019), Gibbs artifact removal (Kellner et al., 2016), susceptibility-induced (Andersson et al., 2003) and eddy current-induced (Andersson & Sotiropoulos, 2016) distortion correction, and cubic interpolation to a 0.5-mm isotropic resolution with MRtrix3 (Tournier et al., 2019) and FSL (Smith et al., 2004).

### 2.4 Cortical column-based analysis of the radial diffusivity

**Fig. 1.**
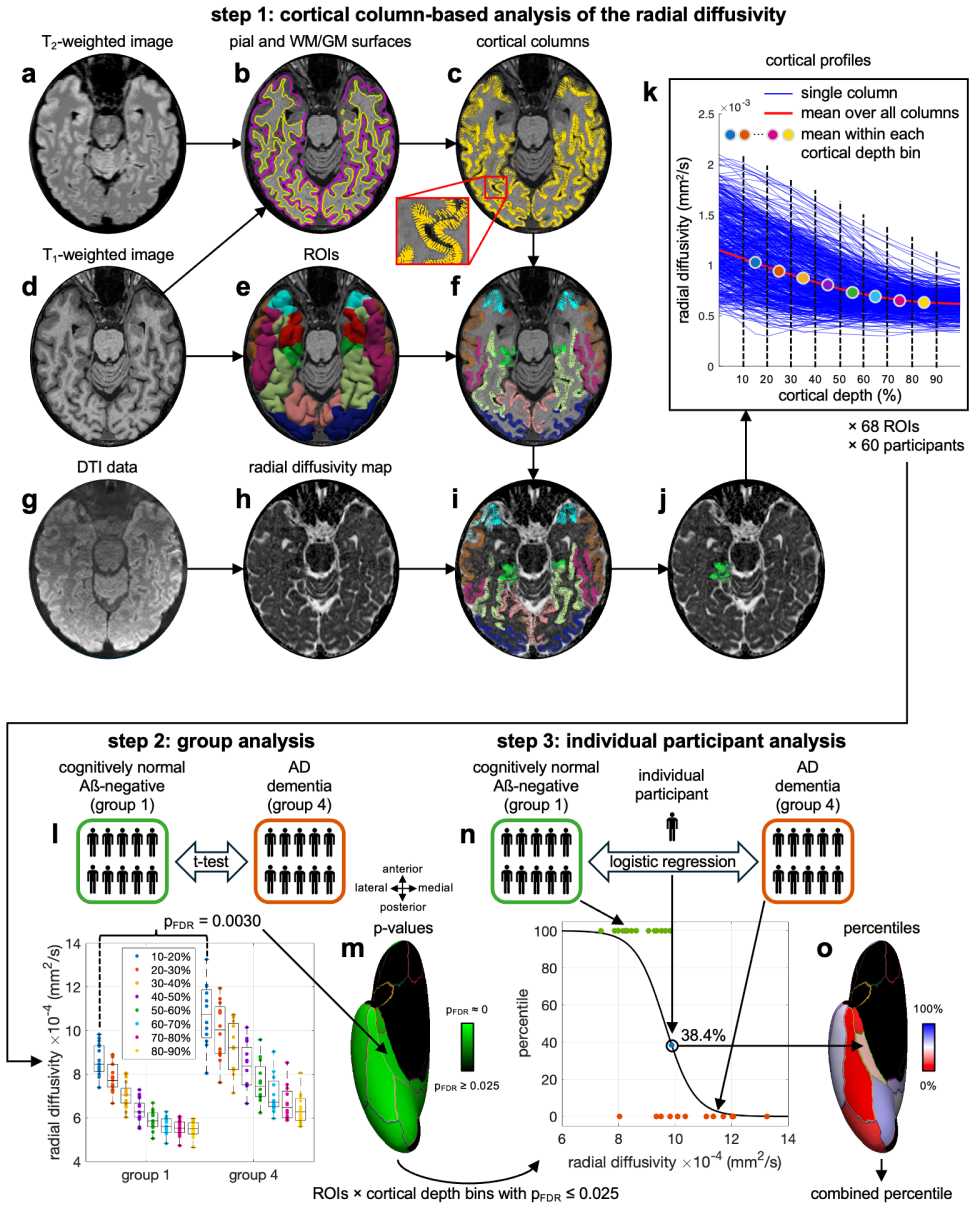
Cortical column-based analysis of the radial diffusivity and statistical analyses. T_1_- and T_2_-weighted anatomical images (a,d) were used to generate pial and WM/GM surface meshes (b) and cortical columns (c) and to parcellate the cortical gray matter into 68 ROIs (e) to which the columns were assigned (f). The dMRI data (g) were used generate a radial diffusivity map (h) and the cortical columns were registered to the diffusion image domain (i). For each ROI (j), the radial diffusivity map was sampled along each column to generate cortical profiles that were then averaged over all columns and within eight cortical depth bins (k). A group analysis was performed between groups 1 and 4 (l), and the p-values were mapped onto a cortical surface (m) to identify all ROIs and cortical depth bins with significant differences in radial diffusivity between these two groups (p_FDR_ ≤ 0.025, after correction for multiple comparisons with false discovery rate (FDR)). For each of these ROI/depth bin combinations, an individual participant analysis comparing each participant with those from groups 1 and 4 was then performed (n) and the resulting percentiles were mapped onto a cortical surface (o). Finally, these percentiles were averaged over the ROI/depth bin combinations with the most significant differences (p_FDR_ ≤ 0.0025) to generate a combined percentile.

A cortical column-based analysis was performed to investigate how the radial diffusivity changes along cortical columns orthogonal to the cortical surface, at different depths ranging from the pial surface to the white matter/gray matter (WM/GM) interface, and within different regions-of-interest (ROIs). Specifically, the T_2_-weighted (Fig. 1a) and T_1_-weighted (Fig. 1d) anatomical images of each participant were coregistered and used to generate pial and WM/GM surface meshes (Fig. 1b) with FreeSurfer (Fischl, 2012). Matching pairs of vertices from these two surface meshes were then connected to generate cortical columns orthogonal to the cortical surface (Fig. 1c). Additionally, the T_1_-weighted anatomical images were used to parcellate the cortical gray matter into 68 ROIs (34 per hemisphere) based on the Desikan–Killiany atlas (Desikan et al., 2006) (Fig. 1e) with the Connectome Mapper (Daducci et al., 2012). The cortical columns were then grouped into these 68 ROIs (Fig. 1f). The preprocessed dMRI data (Fig. 1g) were used to derive a radial diffusivity map (Fig. 1h) with MRtrix3. The b = 0 images were registered to the T_1_-weighted anatomical images with *bbregister* (Greve & Fischl, 2009). Then, the inverse of the transformation matrix was applied to register the cortical columns from the anatomical image domain to the diffusion image domain (Fig. 1i).

For each of the 68 ROIs (Fig. 1j), the radial diffusivity map was sampled along each cortical column at 21 equidistant cortical depths ranging from 0% (pial surface) to 100% (WM/GM interface) to generate cortical profiles of the radial diffusivity (Fig. 1k, blue lines). These cortical profiles were then averaged over all cortical columns within each ROI (Fig. 1k, red line) and further averaged within each of eight cortical depth bins: 10–20%,…, 80–90% (Fig. 1k, circles). The 0–10% and 90–100% cortical depths were excluded to minimize partial volume effects with CSF and white matter, respectively. Thus, radial diffusivity metrics were derived within eight cortical depth bins × 68 ROIs × 60 participants.

### 2.5 Statistical analyses

Two types of statistical analyses were performed. First, a group analysis was performed between group 1 (cognitively normal Aß-negative) and group 4 (AD dementia). Specifically, one-tailed t-tests in both directions were performed between the radial diffusivity of group 1 and the radial diffusivity of group 4, for each of the 8 cortical depth bins and each of the 68 ROIs (Fig. 1l). One-tailed rather than two-tailed t-tests were used to determine not only significant differences in radial diffusivity between these two groups, but also whether the radial diffusivity was higher or lower for one group than for the other. The resulting p-values were corrected for multiple comparisons across ROIs and cortical depth bins via FDR (Rouam, 2013). The statistically significant FDR-corrected p-values (p_FDR_ ≤ 0.025) were then mapped onto an inflated cortical surface (Fig. 1m) for each cortical depth bin.

Second, an individual participant analysis was performed for each of the 60 participants. Specifically, a logistic regression model (sigmoid function) was fit to the radial diffusivity of the participants from groups 1 and 4, and the resulting fit was used along with the radial diffusivity of the participant under test to derive a percentile (Fig. 1n). This procedure was performed for each ROI/depth bin combination with a significantly higher radial diffusivity for group 4 than for group 1 identified in the group analysis (p_FDR_ ≤ 0.025). For each participant, the percentiles were then mapped onto an inflated cortical surface (Fig. 1o) for each cortical depth bin. Finally, these percentiles were averaged (unweighted) over the ROI/depth bin combinations with the most significant differences (p_FDR_ ≤ 0.0025) to yield a combined percentile for each participant. When testing an individual participant from group 1 or group 4, the participant under test was excluded from group 1 or group 4, respectively, before fitting the logistic regression model.

### 2.6 Additional analyses

Secondary analyses were also performed for comparison with the primary analysis described above. Specifically, the analysis was repeated by

1. averaging the percentiles from all ROIs with p_FDR_ ≤ 0.0025, but only within one cortical depth bin at a time (e.g., 10–20%) rather than all of them, to derive the combined percentiles;
2. using a single cortical depth bin of 10–90% (i.e., no cortical depth dependence) rather than eight of them;
3. simply averaging the radial diffusivity over all voxels within each ROI (i.e., voxel-based analysis) rather than sampling it along cortical columns;
4. using three other dMRI metrics (axial diffusivity, mean diffusivity, and fractional anisotropy) rather than the radial diffusivity;
5. using the cortical thickness of each cortical column.

Additionally, the correlation coefficient was calculated between the combined percentiles (from the primary analysis) and the Aß42/40 ratio of the participants.

## 3 Results

### 3.1 Group analysis

**Fig. 2.**
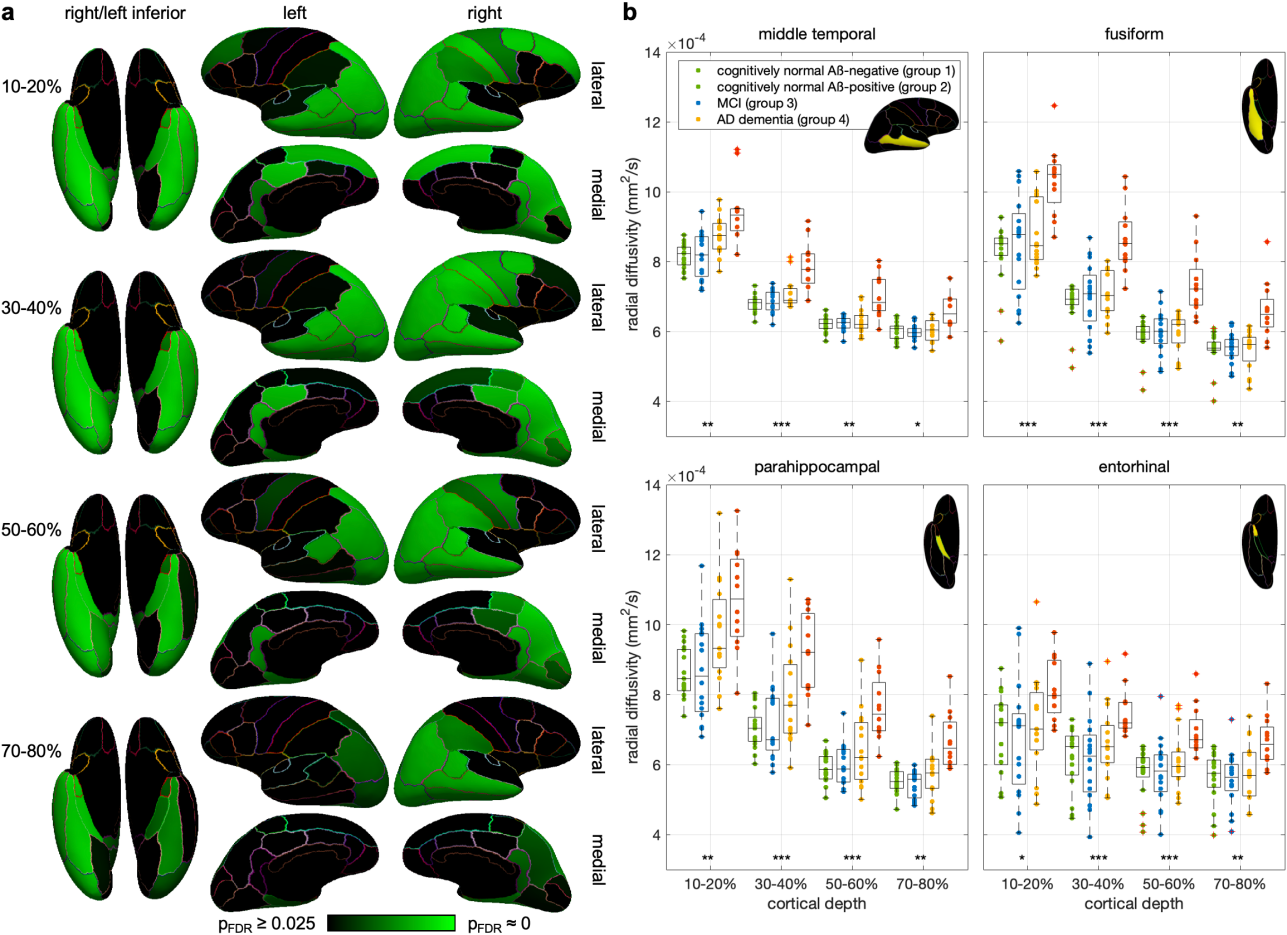
Results of the group analysis. (a) Inferior, lateral, and medial views of an inflated cortical surface showing the ROIs and cortical depth bins with a significantly higher radial diffusivity for group 4 than for group 1 (p_FDR_ ≤ 0.025). (b) Box plots comparing the radial diffusivity of groups 1–4 within each of four cortical depth bins of four representative ROIs with the most significant differences (center line: median, box limits: upper and lower quartiles, whiskers: maximum and minimum, excluding outliers (red crosses), *p_FDR_ ≤ 0.01, **p_FDR_ ≤ 0.005, ***p_FDR_ ≤ 0.0025).

We first report the results of the group analysis to identify the ROI/depth bin combinations with statistically significant (p_FDR_ ≤ 0.025) differences in radial diffusivity between group 1 (cognitively normal Aß-negative) and group 4 (AD dementia). Figure 2a shows that there were 238 ROI/depth bin combinations (out of 68 × 8 = 544 possible combinations) with a significantly higher radial diffusivity for group 4 than for group 1. In contrast, there were no ROI/depth bin combinations with a significantly lower radial diffusivity for group 4. The ROI/depth bin combinations with a significantly higher radial diffusivity for group 4 were not uniformly distributed throughout the brain, but were most prominent in the temporal lobes, which are known to experience the earliest neurodegeneration in AD, followed by the parietal lobes, cingulate cortex, occipital lobes, and frontal lobes, with 91 out of 144 combinations (63.2%), 70 out of 112 (62.5%), 35 out of 80 (43.8%), 24 out of 64 (37.5%), and 18 out of 144 (12.5%), respectively. Additionally, the number of such combinations was the highest in the 20–30% cortical depth bin and progressively decreased in more superficial or deeper cortical depth bins (36, 40, 38, 32, 31, 25, 20, and 16 for 10–20%,…, 80–90%, respectively). Supplementary Table S1 and Supplementary Figure S1a show the p-values for all ROIs and cortical depth bins.

Previous studies have reported an increased diffusivity for individuals with clinically established AD compared with healthy controls within specific regions of the cortical gray matter, including the middle temporal gyrus, fusiform gyrus, parahippocampal gyrus, and entorhinal cortex (Weston et al., 2015). The results of our group analysis are consistent with these studies and show that the radial diffusivity was significantly higher for group 4 than for group 1 within all cortical depth bins of these four representative ROIs known to be affected early by neurodegeneration in AD (Fig. 2b and Supplementary Fig. S2). Other regions with significant differences included the middle frontal, superior parietal, supramarginal, precuneus, inferior parietal, inferior temporal, posterior cingulate, and isthmus cingulate cortices, also consistent with previous studies (Montal et al., 2018; Sun et al., 2024; Weston et al., 2015, 2020). In contrast to these studies performed at a lower resolution, however, our column-based analysis of high-resolution dMRI data further shows a cortical depth dependence of the results, with more significant differences generally seen at cortical depths (~10–60%) corresponding approximately to the middle cortical layers (II–V), where neuronal loss in AD was found to be more pronounced in histological studies (Astillero-Lopez et al., 2022; Gómez-Isla et al., 1996; Thangavel et al., 2008).

### 3.2 Individual participant analysis

We next report the results of the individual participant analysis. The resulting percentiles, either mapped onto cortical surfaces or averaged over the ROI/depth bin combinations with the most significant differences (p_FDR_ ≤ 0.0025), provide an individualized risk assessment of impending cognitive decline for each participant, with high vs. low percentiles indicating that the participant under test had radial diffusivity metrics more similar to those of group 1 vs. group 4, respectively. These 20 ROI/depth bin combinations included 1 in the middle temporal gyrus, 8 in the fusiform gyrus, 5 in the parahippocampal gyrus, and 3 in the entorhinal cortex, all of which are in the temporal lobes. The number of such combinations was the highest in the 30–40% cortical depth bin and progressively decreased in more superficial or deeper cortical depth bins (2, 3, 7, 4, 3, 1, 0, and 0 for 10–20%,…, 80–90%, respectively).

**Fig. 3.**
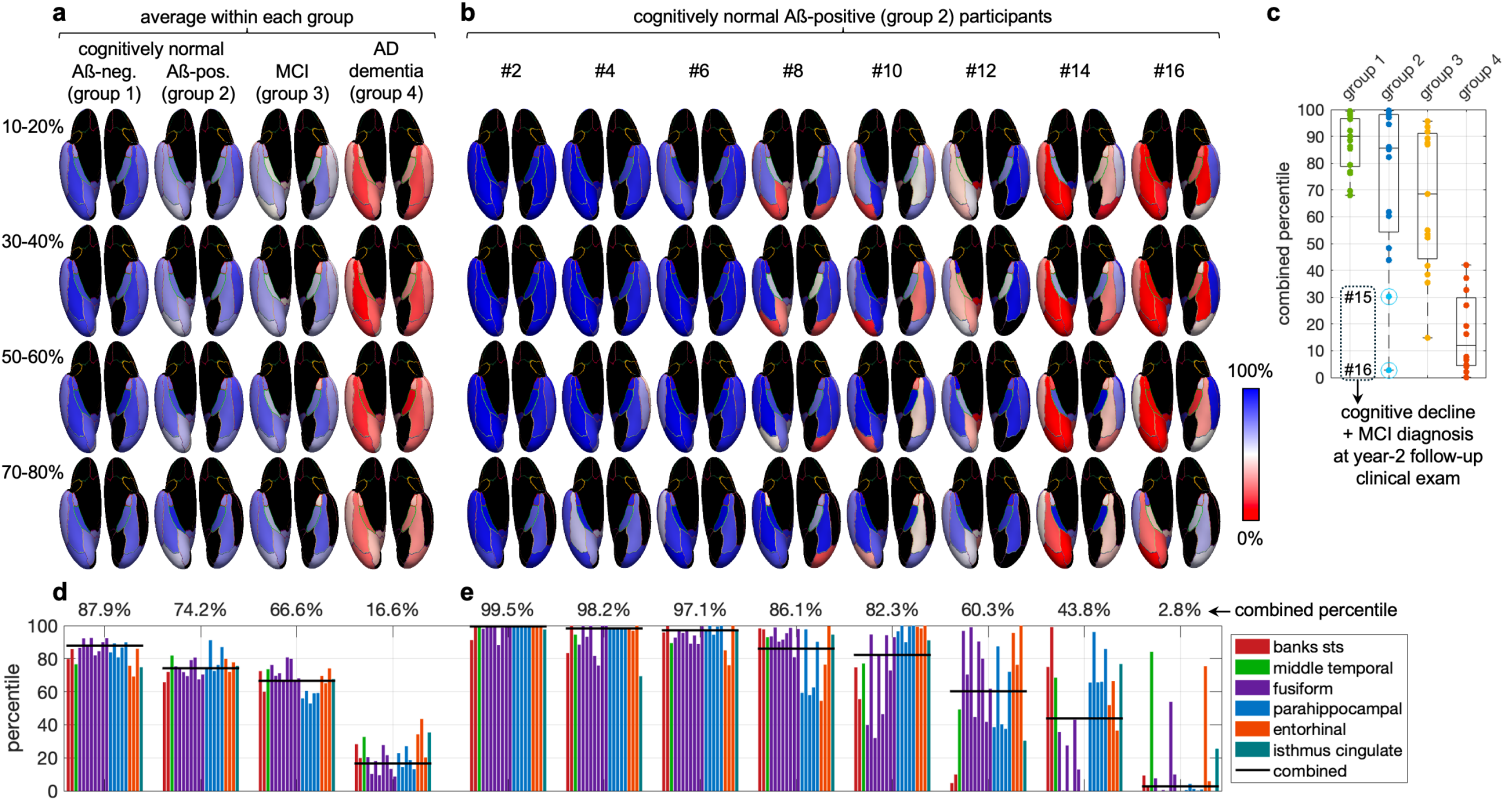
Results of the individual participant analysis. Inferior views of an inflated cortical surface showing the percentiles within each ROI and each of four cortical depth bins, either averaged over all participants within each group (a) or for eight representative participants from group 2 (b). Percentiles were only calculated for ROI/depth bin combinations with a significantly higher radial diffusivity for group 4 than for group 1 identified in the group analysis (p_FDR_ ≤ 0.025, green ROIs in Fig. 2a). The participants from group 2 are ranked by decreasing combined percentiles, which were derived by averaging the percentiles over the ROI/depth bin combinations with the most significant differences (p_FDR_ ≤ 0.0025). Box plots of the combined percentiles (c). Percentiles in each of the ROI/depth bin combinations with p_FDR_ ≤ 0.0025, along with the combined percentiles, either averaged over all participants within each group (d) or for eight representative participants from group 2 (e). STS: superior temporal sulcus.

First, results *averaged over all participants within each group* show progressively decreasing percentiles (i.e., an increasing radial diffusivity) from group 1 to group 4, both for the individual ROIs and cortical depth bins identified in the group analysis (Fig. 3a and 3d) and for the combined percentile (Fig. 3c). This expected downward trend along the AD continuum is consistent with previous studies (Montal et al., 2018; Sun et al., 2024) and validates our methodology at the group level.

Next, results for *individual participants from group 2* (cognitively normal Aß-positive) show a wide range of percentiles, both for individual ROIs and cortical depth bins (Fig. 3b and 3e) and for the combined percentiles (2.8–99.6%), compared with that of group 1 (68.0–99.4%) and group 4 (0.0–42.0%) (Fig. 3c). Some of these participants (e.g., participant 2) had high percentiles closer to those of group 1 (normal controls), whereas others (e.g., participant 16) had low percentiles closer to those of group 4 (AD dementia). Among all participants from group 2 and all ROI/depth bin combinations with p_FDR_ ≤ 0.0025, participant 16 had the lowest percentile in the fusiform and parahippocampal gyri, the second lowest percentile in the entorhinal cortex, and the lowest combined percentile, whereas participant 15 had the third lowest percentile in the fusiform gyrus, the second lowest percentile in the parahippocampal gyrus, the lowest percentile in the entorhinal cortex, and the second lowest combined percentile. Both of these participants were cognitively normal at the time of MRI scanning (baseline visit). At their year-2 follow-up clinical visit, however, they had a cognitive decline with a 5-point and 6-point drop in Montreal Cognitive Assessment (MoCA) score (Nasreddine et al., 2005), respectively, and were diagnosed with MCI. In comparison, the other 14 participants from group 2 generally had higher percentiles in these AD-related brain regions and all 11 of those participants who have so far had a year-2 follow-up clinical examination did not experience any MoCA score decrease of more than 2 points. Such a low conversion rate across a 2-year span is expected for asymptomatic individuals with stage-1 AD. Importantly, the conversion of these two participants with the lowest percentiles in regions associated with AD demonstrates the initial promise of our cortical column-based analysis of the radial diffusivity to predict impending cognitive decline. Supplementary Figures S3–S7 and Supplementary Table S2 show the results for all 60 participants.

### 3.3 Additional analyses

Results derived from each of the 10–20%,…, 50–60% cortical depth bins alone (Supplementary Figs. S1a, S2, S8b-f) or from a single cortical depth bin of 10–90% (Supplementary Figs. S1b, S2, S8j) were similar to the primary results (Supplementary Figs. S1a, S2, S8a) in that the combined percentiles of the two participants from group 2 who had cognitive decline and MCI diagnosis at their follow-up clinical examination (participants 15 and 16) were also the two lowest values within group 2, but the combined percentiles of groups 1 and 4 had wider and/or overlapping ranges. Using only one small cortical depth bin (e.g., 10–20%) leaves out all the ROIs with significant differences from the remaining depth bins, whereas using only one large cortical depth bin (e.g., 10–90%) averages out the diffusivity across all of these cortical depths, including those with no significant differences, both of which can result in a lower performance.

Results derived from a voxel-based analysis (Supplementary Figs. S1c, S2, S8k), which also did not take into account any cortical depth dependence, showed less significant differences, with no ROIs reaching p_FDR_ ≤ 0.0025. Since the cortex is highly convoluted, it cannot be sampled as accurately if using a voxel grid rather than columns orthogonal to the cortical surface and bound by the pial surface and WM/GM interface. These comparisons show that it is better to use a column-based analysis, to sample the columns at different depths, and to take into account all cortical depth bins with significant differences, as in the primary analysis.

Results derived from the mean diffusivity (Supplementary Figs. S9b-S10b) were similar to those derived from the radial diffusivity (Supplementary Figs. S9a-S10a), since the latter accounts for 2/3 of the mean diffusivity, but the combined percentiles of groups 1 and 4 had wider ranges. Results derived from the axial diffusivity and fractional anisotropy (Supplementary Figs. S9c,d-S10c,d) were different in that the combined percentiles of participants 15 and 16 from group 2 were not the two lowest values within that group. For the axial diffusivity, the ROI/depth bin combinations with the most significant differences (p_FDR_ ≤ 0.0025) included the fusiform and parahippocampal gyri (as for the radial and mean diffusivity), but also the postcentral and inferior temporal gyri. For the fractional anisotropy, they did not overlap with any of the AD-relevant regions observed with the diffusivity metrics, but were instead in the precuneus cortex, superior temporal gyrus, pericalcarine cortex, and cuneus cortex.

Results derived from the cortical thickness (Supplementary Figs. S9e–S10e) showed multiple ROIs with a significantly smaller thickness for group 4 than for group 1, particularly in the temporal and parietal lobes, as expected from previous studies (Montal et al., 2018; Sun et al., 2024). However, the combined percentiles of participants 15 and 16 from group 2 were not the two lowest values within that group. While the diffusivity reflects microstructural changes, the cortical thickness reflects macrostructural changes occurring at later symptomatic stages (Weston et al., 2015) and may not accurately predict risk of impending cognitive decline among the cognitively normal participants from group 2, who had no significant differences in cortical thickness compared with those from group 1. Additionally, while the diffusivity is internally normalized, results derived from the cortical thickness may be confounded by individual differences across participants, even though previous studies found that cortical thickness was not highly correlated with head size (Schwarz et al., 2016) and is thus typically not normalized, as opposed to volume measurements.

Finally, there was a weak correlation between the combined percentiles (from the primary analysis; Fig. 3c) and the Aß42/40 ratio across all four groups combined (R^2^ = 0.207) because all participants from group 1 were Aß-negative and had high percentiles, whereas all participants from group 4 with a known Aß status were Aß-positive and had low percentiles (Supplementary Fig. S11). However, that correlation was very weak within group 2 (R^2^ = 0.055). In particular, participants 15 and 16 from group 2 had the 6^th^ and 10^th^ lowest Aß42/40 ratios among the 16 participants from that group, showing that these ratios did not predict impending cognitive decline as the combined percentiles did.

## 4 Discussion and Conclusions

To detect early microstructural neurodegeneration before the onset of cognitive symptoms, we used a cortical column-based analysis of high-resolution dMRI data to investigate how the radial diffusivity changes along cortical columns, across different depths and ROIs. Our group analysis identified ROI/depth bin combinations with a significantly higher radial diffusivity for group 4 (AD dementia) than for group 1 (cognitively normal Aß-negative) in regions known to be affected by neurodegeneration in AD. Moreover, the radial diffusivity progressively increased in these regions from group 1 to group 4, thus validating its dependence on the disease progression and cognitive decline. Most importantly, our analysis in individual participants from group 2 (cognitively normal Aß-positive, i.e., stage-1 AD, at the time of MRI scanning) provided individualized risk assessments of impending cognitive decline, as confirmed by follow-up clinical examinations. Such a finding could be highly valuable to help plan for preventative measures as well as early treatments to delay the onset or slow the progression of symptoms.

In contrast to previous studies performed with a lower resolution and a voxel-based analysis, our cortical column-based analysis of high-resolution dMRI data was able to identify differences in radial diffusivity not only within specific ROIs, but also within specific cortical depth bins, which have selectively vulnerable neuronal populations (Astillero-Lopez et al., 2022; Gómez-Isla et al., 1996; Thangavel et al., 2008). Our results showed that it was better to use only ROI/depth bin combinations with significant differences rather than a voxel-based analysis or a column-based analysis that did not take into account any cortical depth dependence.

Partial volume effects between gray matter and either CSF or white matter, which have a higher or lower diffusivity, respectively, could have contributed to the progressively decreasing radial diffusivity from the pial surface to the WM/GM interface seen in all four groups (Fig. 2b and Supplementary Fig. 2), although an ex vivo dMRI study (Kleinnijenhuis et al., 2013) performed with a 0.3-mm isotropic resolution also found a similar cortical depth-dependent variation in diffusivity, consistent with an increased density of myelinated axons from superficial to deep cortical layers. Additionally, cortical atrophy in the participants from group 4, resulting in more partial volume effects with CSF, could also have contributed to the higher radial diffusivity seen in that group than in group 1. However, the number of ROI/depth bin combinations with the most significant differences (p_FDR_ ≤ 0.0025) was the highest in the 30–40% cortical depth bin. Furthermore, results derived from the radial diffusivity, reflecting microstructural changes, provided a better risk prediction of impending cognitive decline among the cognitively normal participants from group 2 with no cortical atrophy, as compared with results derived from the cortical thickness, reflecting macrostructural changes occurring at later symptomatic stages (Weston et al., 2015).

While the initial results obtained with our novel methodology are promising, our study had a relatively limited sample size and length (2 years) of clinical follow-up after baseline MRI scanning. These limitations can be addressed in the future as we continue to cognitively evaluate participants yearly and to enroll new participants as part of our ongoing longitudinal study. Because of the limited number of participants in each group, we were not able to further stratify the participants by age, which would be desirable given that age is the largest risk factor for neurodegenerative diseases and there is often interplay between the mechanisms underlying those diseases and that of normal aging (Pandya & Patani, 2021) itself. Additionally, there was a high proportion of females in groups 1, 2, and 4 (82%, 69%, and 83%). Since sex differences in dMRI metrics have been reported in both normal aging (Ng et al., 2013) and AD (Bergamino et al., 2022), a larger number of males are needed to generalize the results to larger populations. A larger sample size would also enable further stratification in groups 1 and 2 based on other risk factors for AD, such as the ε4 allele of the apolipoprotein E gene (APOE4) or a family history of AD, to investigate the influence of these risk factors on early neurodegeneration with our cortical column-based analysis of the radial diffusivity. Finally, a finer cortical parcellation of the cortical gray matter into smaller ROIs could be used to achieve a higher spatial specificity (Ma et al., 2023) and help further improve the clinical utility of our methodology. Overall, the promising results obtained in this study provide a strong justification for a more systematic long-term trial with larger sample sizes.

## Supplementary Material

Supplementary Material

## Data Availability

Data supporting the findings of this study can be made available upon reasonable request to the corresponding author. The data analysis was performed with common publicly available software: MRtrix3 (https://www.mrtrix.org), FSL (https://fsl.fmrib.ox.ac.uk), and FreeSurfer (https://surfer.nmr.mgh.harvard.edu).
