# Supplementary material for: Predicting risk of impending cognitive decline in asymptomatic individuals with early Alzheimer’s disease: Insights from cortical diffusion MRI
Source: Imaging Neurosci (Camb). 2025 Nov 24;3:IMAG.a.1037. doi: 10.1162/IMAG.a.1037 (PMC12645123; doi:10.1162/IMAG.a.1037)
Supplement: Supplementary Material [file IMAG.a.1037_supp.pdf]

Supplementary Materials

Lobe	ROI	10-20% left	10-20% right	20-30% left	20-30% right	30-40% left	30-40% right	40-50% left	40-50% right	50-60% left	50-60% right	60-70% left	60-70% right	70-80% left	70-80% right	80-90% left	80-90% righ'	10-90% left	10-90% right	voxel left	voxel right
frontal lobe	frontalpole	0.1727	0.0762	0.1905	0.0701	0.2130	0.0670	0.2361	0.0670	0.2485	0.0670	0.2431	0.0666	0.2235	0.0657	0.1982	0.0638	0.2017	0.0591	0.2421	0.0481
	medialorbitofrontal	0.1169	0.1274	0.1143	0.0973	0.1312	0.0760	0.1719	0.0701	0.2295	0.0760	0.2926	0.0891	0.3392	0.1017	0.3602	0.1065	0.1929	0.0637	0.1324	0.0282
	lateralorbitofrontal	0.0748	0.0504	0.0531	0.0670	0.0450	0.0884	0.0448	0.1066	0.0465	0.1131	0.0469	0.1064	0.0445	0.0907	0.0401	0.0758	0.0354	0.0691	0.0430	0.0430
	parsorbitalis	0.1329	0.0947	0.1065	0.0793	0.0905	0.0925	0.0813	0.1143	0.0748	0.1242	0.0686	0.1169	0.0623	0.1017	0.0550	0.0847	0.0710	0.0803	0.0560	0.0560
	parstriangularis	0.1065	0.0318	0.1218	0.0554	0.1283	0.0831	0.1251	0.1090	0.1120	0.1209	0.0894	0.1131	0.0670	0.0907	0.0475	0.0670	0.0779	0.0558	0.0545	0.0324
	rostralmiddlefrontal	0.0139	0.0053	0.0224	0.0113	0.0335	0.0217	0.0477	0.0344	0.0635	0.0492	0.0733	0.0635	0.0746	0.0709	0.0686	0.0729	0.0299	0.0231	0.0218	0.0171
	caudalmiddlefrontal	0.0243	0.0056	0.0229	0.0048	0.0245	0.0066	0.0299	0.0138	0.0378	0.0330	0.0495	0.0666	0.0621	0.1060	0.0679	0.1380	0.0190	0.0090	0.0114	0.0086
	parsopercularis	0.1275	0.0644	0.0993	0.0550	0.0842	0.0477	0.0841	0.0430	0.0925	0.0424	0.1035	0.0456	0.1027	0.0531	0.0894	0.0616	0.0691	0.0312	0.0530	0.0263
	superiorfrontal	0.0076	0.0048	0.0138	0.0092	0.0242	0.0186	0.0392	0.0335	0.0603	0.0531	0.0816	0.0699	0.1017	0.0768	0.1093	0.0760	0.0270	0.0198	0.0219	0.0130
parietal lobe	paracentral	0.4430	0.0301	0.2054	0.0194	0.0815	0.0122	0.0497	0.0094	0.0531	0.0113	0.0760	0.0219	0.1181	0.0459	0.1758	0.0898	0.1131	0.0086	0.0944	0.0141
	precentral	0.0380	0.0129	0.0287	0.0103	0.0223	0.0083	0.0186	0.0080	0.0186	0.0107	0.0224	0.0180	0.0296	0.0301	0.0389	0.0424	0.0154	0.0045	0.0114	0.0051
	postcentral	0.0473	0.0048	0.0352	0.0043	0.0291	0.0043	0.0279	0.0067	0.0321	0.0150	0.0445	0.0332	0.0670	0.0584	0.0925	0.0816	0.0261	0.0058	0.0192	0.0051
	superiorparietal	0.0043	0.0039	0.0043	0.0039	0.0048	0.0041	0.0055	0.0043	0.0075	0.0053	0.0115	0.0075	0.0186	0.0116	0.0287	0.0175	0.0045	0.0031	0.0055	0.0041
	supramarginal	0.0226	0.0107	0.0228	0.0056	0.0243	0.0043	0.0288	0.0045	0.0369	0.0060	0.0492	0.0092	0.0644	0.0118	0.0724	0.0127	0.0231	0.0031	0.0218	0.0037
	precuneus	0.1150	0.0032	0.0623	0.0030	0.0378	0.0032	0.0282	0.0035	0.0248	0.0043	0.0271	0.0080	0.0317	0.0166	0.0345	0.0275	0.0283	0.0022	0.0192	0.0037
	inferiorparietal	0.0043	0.0043	0.0043	0.0037	0.0048	0.0035	0.0055	0.0035	0.0073	0.0035	0.0105	0.0040	0.0150	0.0048	0.0195	0.0059	0.0045	0.0018	0.0051	0.0031
temporal lobe	superiortemporal	0.0230	0.0330	0.0301	0.0224	0.0391	0.0224	0.0550	0.0301	0.0833	0.0430	0.1354	0.0576	0.2009	0.0659	0.2178	0.0625	0.0399	0.0216	0.0430	0.0144
	bankssts	0.0043	0.0035	0.0043	0.0029	0.0043	0.0023	0.0048	0.0023	0.0084	0.0029	0.0193	0.0039	0.0335	0.0053	0.0378	0.0078	0.0031	0.0015	0.0077	0.0026
	middletemporal	0.0032	0.0037	0.0035	0.0027	0.0048	0.0023	0.0104	0.0027	0.0241	0.0035	0.0483	0.0043	0.0750	0.0053	0.0865	0.0052	0.0067	0.0015	0.0192	0.0031
	temporalpole	0.1220	0.0550	0.1098	0.0497	0.1087	0.0576	0.1150	0.0680	0.1259	0.0741	0.1500	0.0690	0.1905	0.0565	0.2294	0.0433	0.1206	0.0389	0.1178	0.0192
	transversetemporal	0.2894	0.6180	0.2037	0.3399	0.1592	0.1433	0.1509	0.0746	0.1773	0.0504	0.2359	0.0430	0.3165	0.0424	0.3811	0.0450	0.2027	0.1046	0.2089	0.0782
	inferiortemporal	0.0035	0.0043	0.0043	0.0040	0.0064	0.0039	0.0112	0.0040	0.0217	0.0043	0.0377	0.0050	0.0587	0.0073	0.0758	0.0105	0.0090	0.0031	0.0202	0.0037
	fusiform	0.0022	0.0008	0.0022	0.0008	0.0023	0.0009	0.0027	0.0015	0.0035	0.0022	0.0050	0.0027	0.0104	0.0035	0.0182	0.0041	0.0016	0.0007	0.0058	0.0026
	parahippocampal	0.0041	0.0030	0.0037	0.0023	0.0035	0.0018	0.0035	0.0015	0.0043	0.0018	0.0075	0.0023	0.0128	0.0032	0.0179	0.0035	0.0017	0.0007	0.0037	0.0026
	entorhinal	0.0075	0.0063	0.0107	0.0035	0.0126	0.0024	0.0132	0.0022	0.0139	0.0023	0.0150	0.0032	0.0160	0.0042	0.0173	0.0050	0.0077	0.0015	0.0114	0.0031
occipital lobe	lateraloccipital	0.0035	0.0029	0.0037	0.0029	0.0043	0.0030	0.0050	0.0032	0.0072	0.0035	0.0107	0.0041	0.0152	0.0048	0.0217	0.0063	0.0041	0.0017	0.0051	0.0026
	pericalcarine	0.1198	0.0288	0.0993	0.0192	0.0748	0.0126	0.0550	0.0112	0.0421	0.0160	0.0372	0.0282	0.0378	0.0403	0.0411	0.0473	0.0394	0.0067	0.0430	0.0114
	lingual	0.0701	0.0035	0.0565	0.0035	0.0435	0.0037	0.0352	0.0048	0.0319	0.0084	0.0330	0.0147	0.0377	0.0228	0.0450	0.0312	0.0312	0.0035	0.0202	0.0051
	cuneus	0.1931	0.0107	0.1251	0.0075	0.0911	0.0065	0.0762	0.0075	0.0733	0.0107	0.0742	0.0160	0.0753	0.0230	0.0742	0.0301	0.0748	0.0053	0.0481	0.0091
	insula	0.0590	0.0252	0.0445	0.0218	0.0389	0.0256	0.0369	0.0335	0.0351	0.0430	0.0330	0.0577	0.0308	0.0783	0.0319	0.1065	0.0261	0.0231	0.0192	0.0192
cingulate	rostralanteriorcingulate	0.0541	0.1982	0.0854	0.1641	0.1290	0.1242	0.1905	0.0975	0.2466	0.0853	0.2858	0.0813	0.3143	0.0813	0.3485	0.0860	0.1604	0.0820	0.1324	0.1178
	caudalanteriorcingulate	0.0229	0.2220	0.0229	0.2054	0.0335	0.1953	0.0587	0.1811	0.0925	0.1500	0.1274	0.1093	0.1500	0.0762	0.1627	0.0635	0.0399	0.1211	0.0341	0.0198
	posteriorcingulate	0.0043	0.0333	0.0035	0.0116	0.0050	0.0051	0.0150	0.0056	0.0421	0.0155	0.0905	0.0430	0.1685	0.0800	0.2520	0.1131	0.0175	0.0058	0.0114	0.0051
	isthmuscingulate	0.0107	0.0035	0.0043	0.0027	0.0038	0.0024	0.0043	0.0028	0.0072	0.0035	0.0171	0.0057	0.0374	0.0129	0.0644	0.0245	0.0049	0.0016	0.0128	0.0051

Supplementary Table S1. FDR-corrected p-values from the group analysis for: each of the 10-20%, …, 80-90% cortical depth bins, a single cortical depth bin of 10-90%, and a voxel-based analysis. ROIs and/or cortical depth bins with a significantly higher radial diffusivity for group 4 (AD dementia) compared to group 1 (cognitively normal Aß-negative) are highlighted in dark blue ($p_{FDR} \leq 0.0025$) or light blue ($0.0025 < p_{FDR} \leq 0.025$).

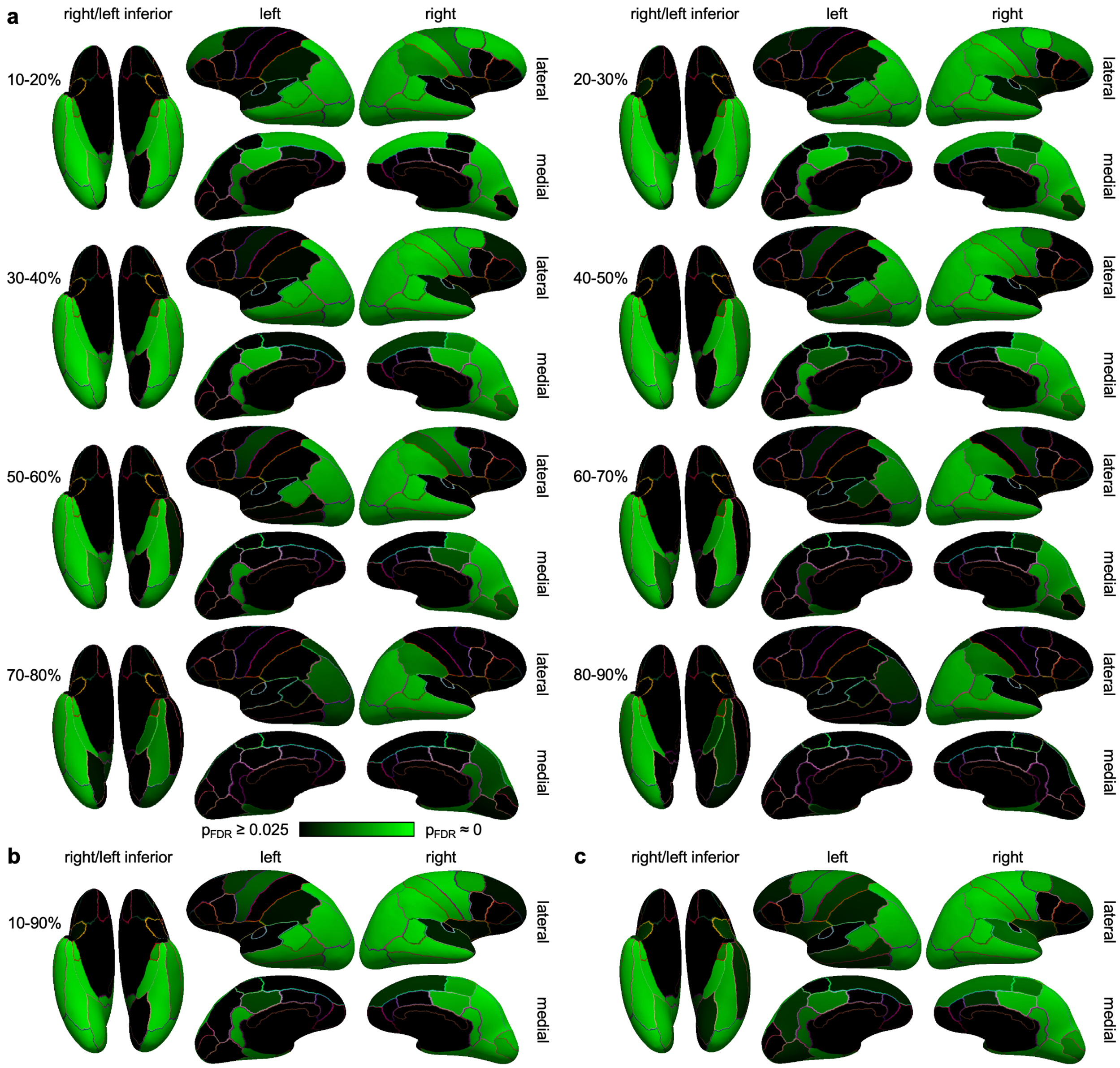

Supplementary Fig. S1. Inferior, lateral, and medial views of an inflated cortical surface showing the ROIs and/or cortical depth bins with a significantly higher radial diffusivity for group 4 compared to group 1 ($p_{FDR} \leq 0.025$) for: (**a**) each of the 10-20%, …, 80-90% cortical depth bins (also in Fig. 2a), (**b**) a single cortical depth bin of 10-90%, and (**c**) a voxel-based analysis. The number of ROIs with $p_{FDR} \leq 0.025$ were 36, 40, 38 , 32, 31, 25, 20, 16, and 38 for the 10-20%, …, 80-90%, and 10-90% cortical depth bins, respectively, and 46 for the voxel-based analysis. There were no ROIs with a significantly lower radial diffusivity for group 4 compared to group 1.

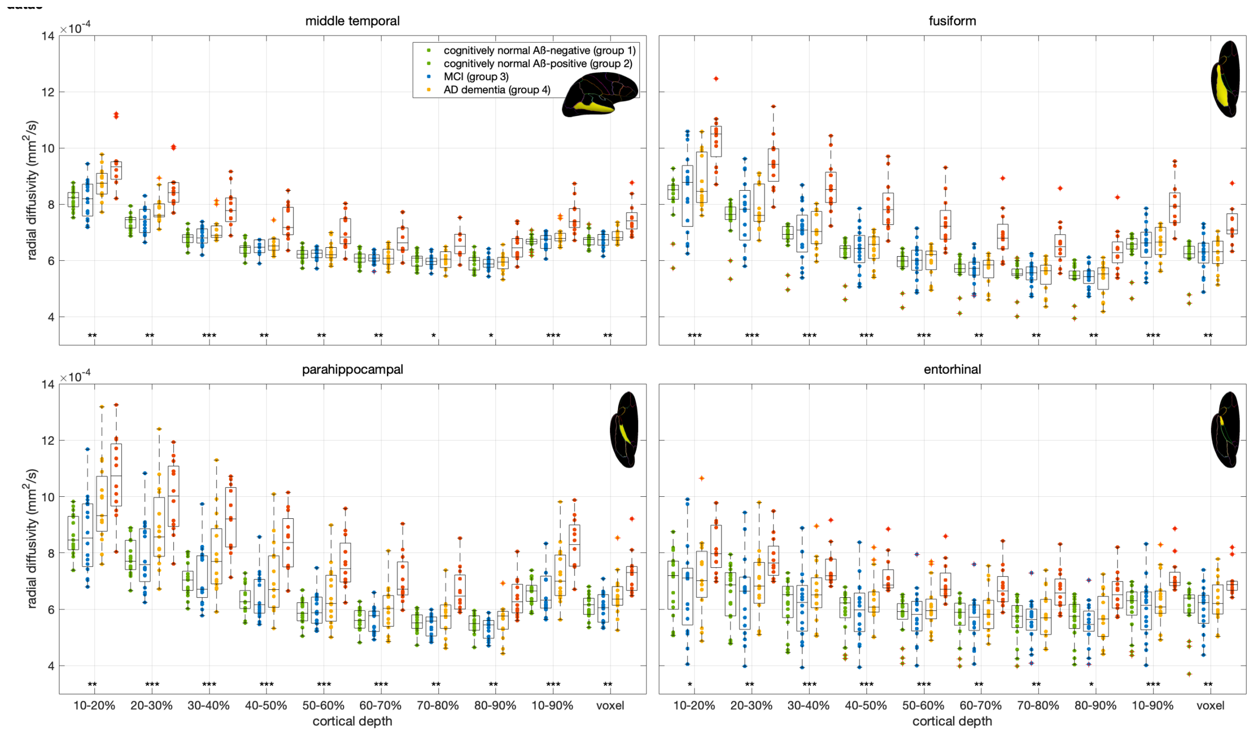

Supplementary Fig. S2. Box plots comparing the radial diffusivity of groups 1–4 for: each of the 10-20%, …, 80-90% cortical depth bins (also in Fig. 2b), a single cortical depth bin of 10-90%, and a voxel-based analysis, for four representative ROIs with the most significant differences between group 1 and group 4 (center line: median, box limits: upper and lower quartiles, whiskers: maximum and minimum, excluding outliers (red crosses), *: $p_{FDR} \leq 0.01$, **: $p_{FDR} \leq 0.005$, ***: $p_{FDR} \leq 0.0025$).

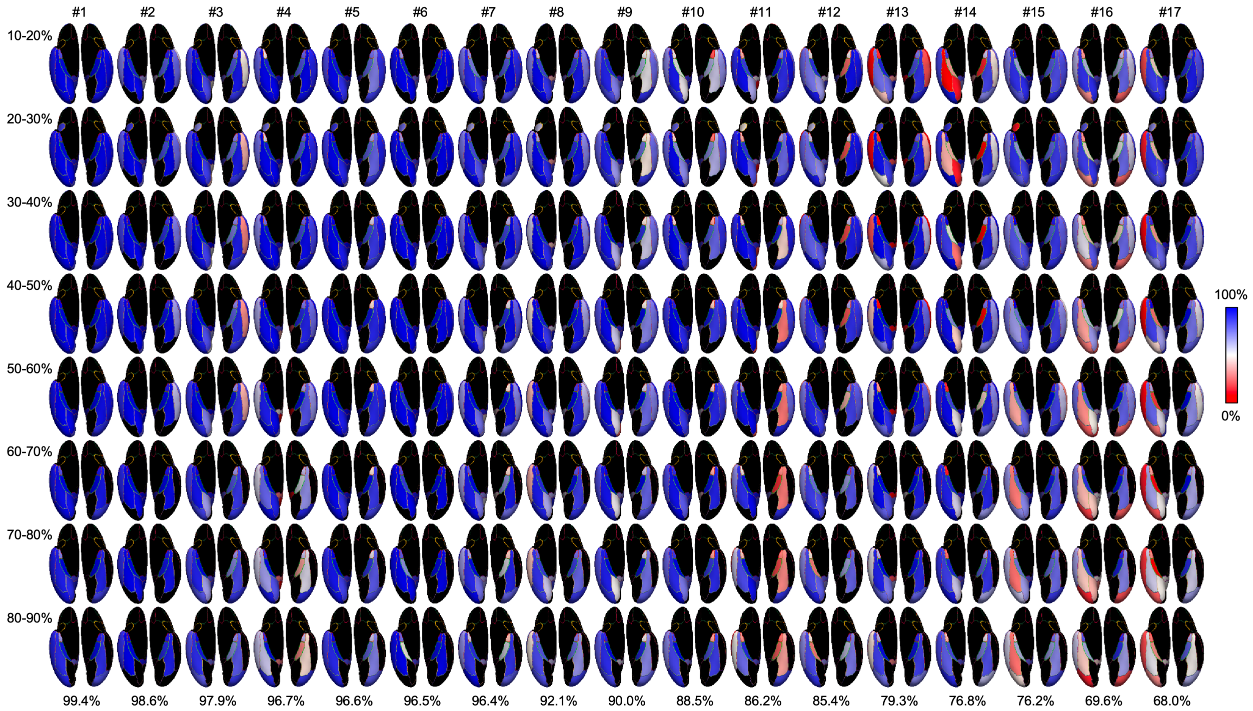

Supplementary Fig. S3. Results of the individual participant analysis for all 17 participants from group 1 (cognitively normal Aß-negative). Inferior views of an inflated cortical surface showing the percentiles within each ROI and each cortical depth bin. The participants are ranked by decreasing combined percentiles, which are shown at the bottom.

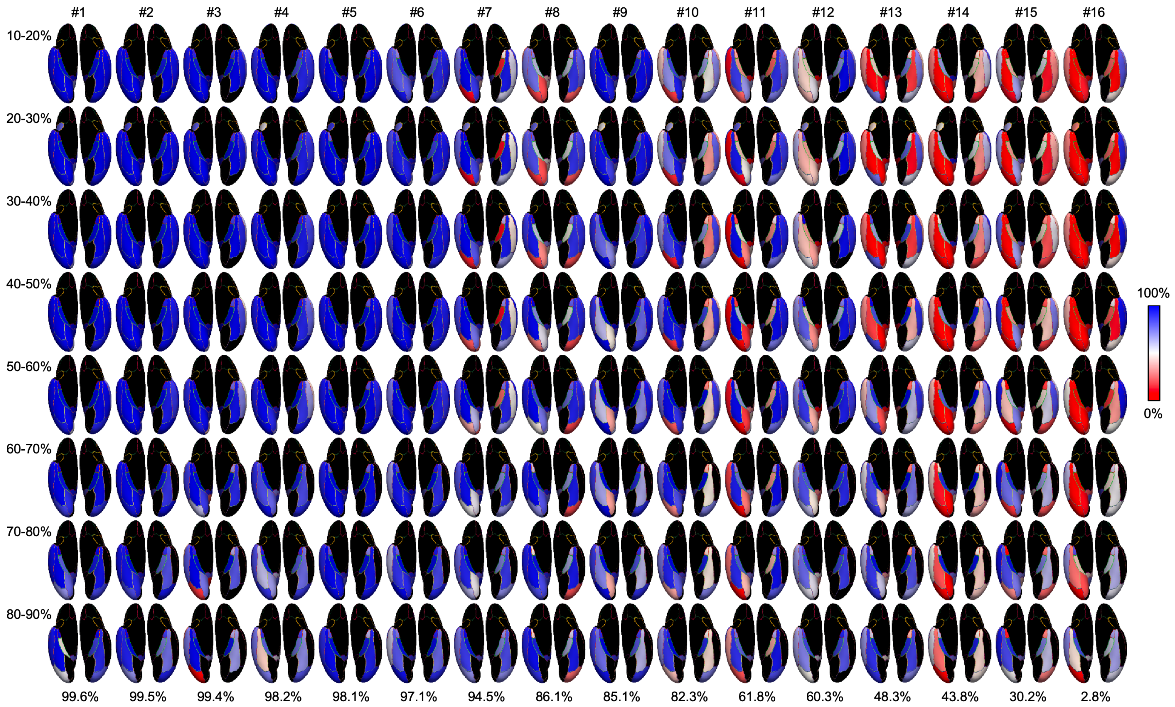

Supplementary Fig. S4. Results of the individual participant analysis for all 16 participants from group 2 (cognitively normal Aß-positive). Inferior views of an inflated cortical surface showing the percentiles within each ROI and each cortical depth bin. The participants are ranked by decreasing combined percentiles, which are shown at the bottom.

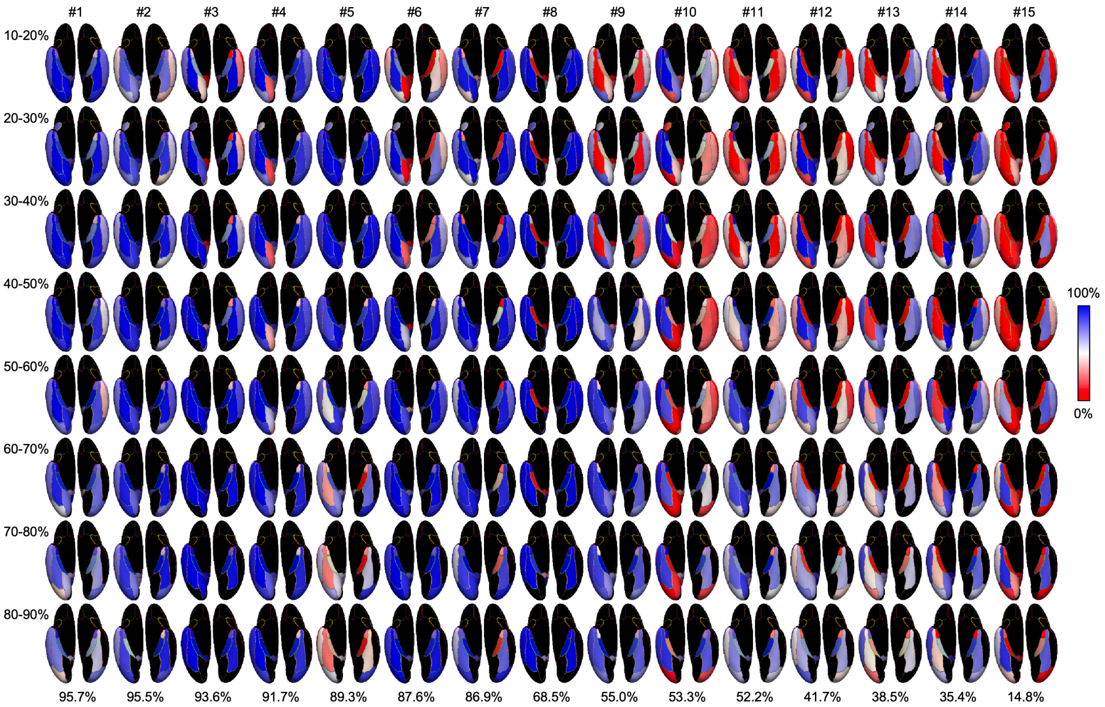

Supplementary Fig. S5. Results of the individual participant analysis for all 15 participants from group 3 (MCI). Inferior views of an inflated cortical surface showing the percentiles within each ROI and each cortical depth bin. The participants are ranked by decreasing combined percentiles, which are shown at the bottom.

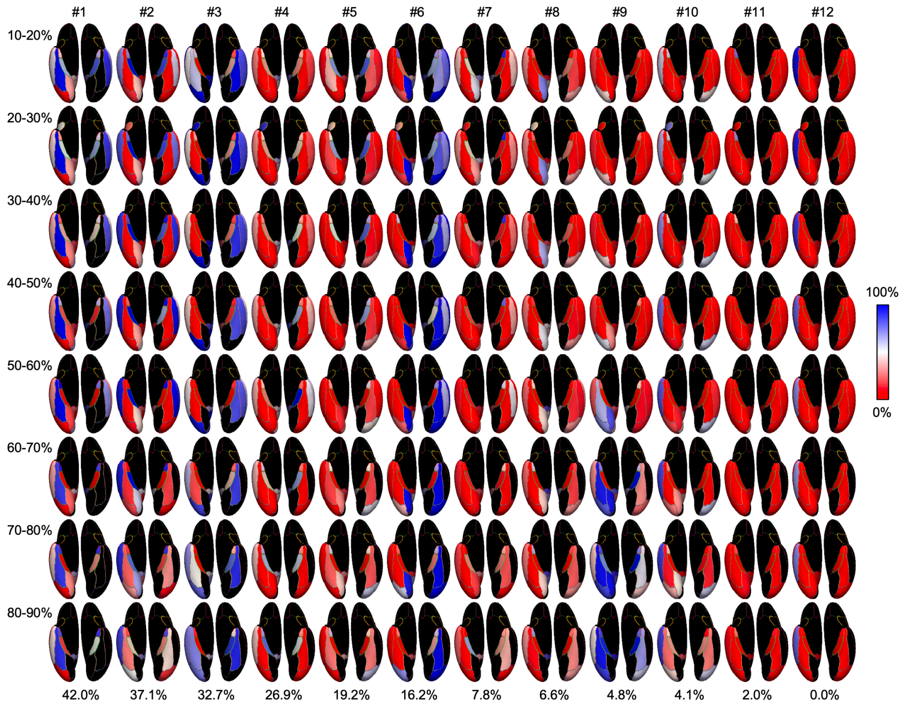

Supplementary Fig. S6. Results of the individual participant analysis for all 12 participants from group 4 (AD dementia). Inferior views of an inflated cortical surface showing the percentiles within each ROI and each cortical depth bin. The participants are ranked by decreasing combined percentiles, which are shown at the bottom.

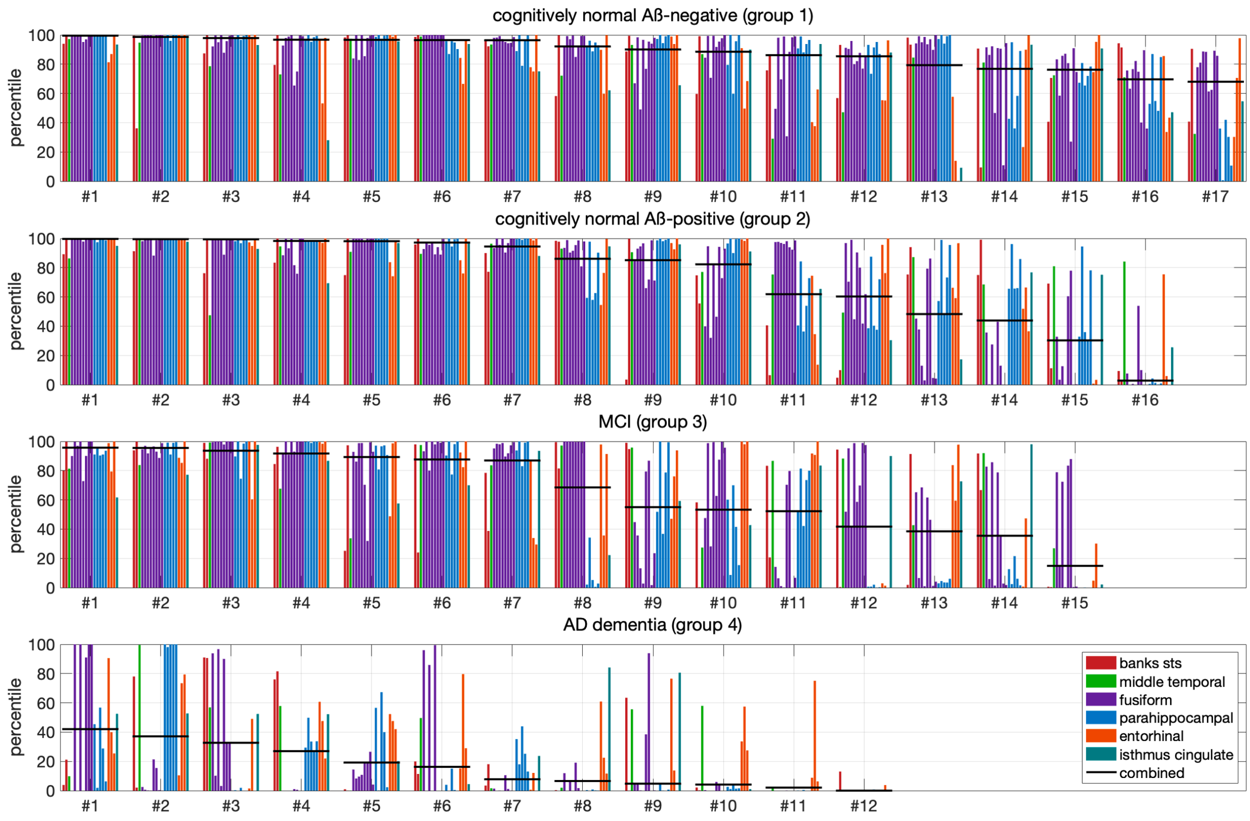

Supplementary Fig. S7. Additional results of the individual participant analysis for all 60 participants. Percentiles in each of the ROI/depth bin combinations with $p_{FDR} \leq 0.0025$, along with the combined percentiles. The participants within each of the four groups are ranked by decreasing combined percentiles. sts: superior temporal sulcus.

	Cognitively normal Aß-negative (group 1)					Cognitively normal Aß-positive (group 2)					MCI (group 3)					AD dementia (group 4)				
**Participant**	**Age**	**Sex**	**Aß**	**f/u**	**Combined percentile**	**Age**	**Sex**	**Aß**	**f/u**	**Combined percentile**	**Age**	**Sex**	**Aß**	**f/u**	**Combined percentile**	**Age**	**Sex**	**Aß**	**f/u**	**Combined percentile**
#1	70.2	F	-	Y	99.4%	56.4	M	+	Y	99.6%	67.9	F	?	N	95.7%	52.1	M	+	Y	42.0%
#2	70.4	M	-	Y	98.6%	56.7	F	+	Y	99.5%	79.4	F	?	N	95.5%	54.9	F	+	Y	37.1%
#3	64.6	F	-	N	97.9%	63.3	M	+	Y	99.4%	66.5	M	?	N	93.6%	78.7	F	?	Y	32.7%
#4	73.4	F	-	Y	96.7%	61.8	F	+	Y	98.2%	58.3	M	-	Y	91.7%	66.6	F	?	N	26.9%
#5	67.2	F	-	Y	96.6%	53.9	F	+	N	98.1%	54.5	F	-	Y	89.3%	68.6	M	+	Y	19.2%
#6	70.0	F	-	N	96.5%	77.7	F	+	Y	97.1%	75.0	M	+	Y	87.6%	76.4	F	+	N	16.2%
#7	65.9	F	-	Y	96.4%	52.2	F	+	N	94.5%	58.6	M	+	Y	86.9%	79.1	F	?	Y	7.8%
#8	51.8	F	-	Y	92.1%	73.8	M	+	Y	86.1%	73.8	M	?	N	68.5%	67.0	F	?	N	6.6%
#9	62.5	F	-	N	90.0%	67.9	F	+	N	85.1%	68.8	M	+	Y	55.0%	81.0	F	+	N	4.8%
#10	77.7	F	-	N	88.5%	72.0	M	+	Y	82.3%	76.7	M	+	Y	53.3%	73.8	F	?	Y	4.1%
#11	72.2	M	-	Y	86.2%	72.3	F	+	Y	61.8%	72.3	M	?	N	52.2%	63.3	F	?	N	2.0%
#12	65.8	M	-	Y	85.4%	72.4	F	+	Y	60.3%	73.2	M	?	N	41.7%	72.3	F	+	Y	0.0%
#13	76.8	F	-	Y	79.3%	63.2	F	+	Y	48.3%	68.2	F	+	Y	38.5%					
#14	73.1	F	-	Y	76.8%	78.7	M	+	Y	43.8%	75.6	F	+	Y	35.4%					
#15	68.7	F	-	N	76.2%	74.4	F	+	Y	30.2%	79.1	M	?	N	14.8%					
#16	72.3	F	-	Y	69.6%	77.1	F	+	Y	2.8%										
#17	72.5	F	-	Y	68.0%															
mean ± SD	69.1 ± 6.1 years					67.1 ± 8.9 years					69.9 ± 7.7 years					69.5 ± 9.3 years				
F / M		82% / 18%					69% / 31%					33% / 67%					83% / 17%			
+ / - / ?			0% / 100% / 0%					100% / 0% / 0%					40% / 13% / 47%					50% / 0% / 50%		
Y / N				71% / 29%					81% / 19%					53% / 47%					58% / 42%	
mean					87.9%					74.2%					66.6%					16.6%

age group difference (two-tailed t-tests): p = 0.46 (groups 1 vs. 2), 0.77 (1 vs. 3), 0.91 (1 vs. 4), 0.37 (2 vs. 3), 0.51 (2 vs. 4), 0.91 (3 vs. 4)

sex group difference (two-tailed χ^2 tests): p = 0.36 (groups 1 vs. 2), 0.005 (1 vs. 3), 0.95 (1 vs. 4), 0.049 (2 vs. 3), 0.38 (2 vs. 4), 0.009 (3 vs. 4)

Supplementary Table S2. Demographics and results of the individual participant analysis for all 60 participants. The combined percentiles are averaged over the ROI/depth bin combinations with the most significantly higher radial diffusivity for group 4 compared to group 1 ($p_{FDR} \leq 0.0025$). The participants within each of the four groups are ranked by decreasing combined percentiles. f/u: year-2 follow-up clinical exam (Y: yes, N: not yet).

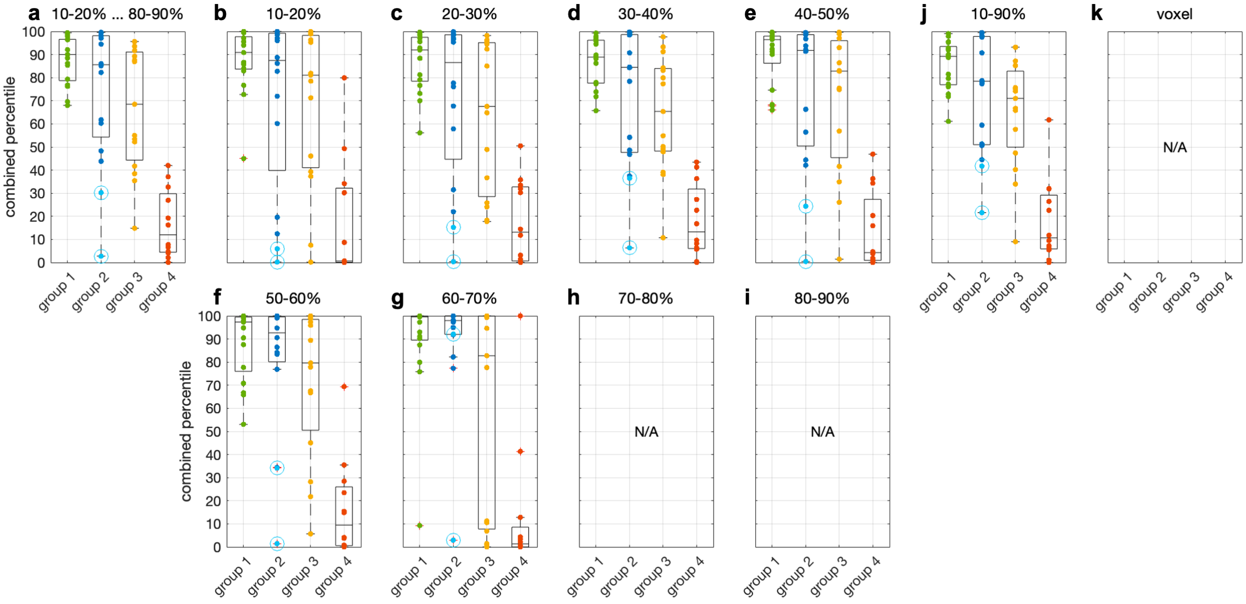

Supplementary Fig. S8. Box plots of combined percentiles derived from the radial diffusivity: (**a**) averaged over the ROI/depth bin combinations with the most significantly higher radial diffusivity for group 4 compared to group 1 ($p_{FDR} \leq 0.0025$) (i.e., same as Fig. 3c); (**b–i**) averaged over the ROIs with $p_{FDR} \leq 0.0025$, but only within one cortical depth bin at a time; (**j**) averaged over the ROIs with $p_{FDR} \leq 0.0025$, but within a single cortical depth bin of 10-90%, or (**k**) averaged over the ROIs with $p_{FDR} \leq 0.0025$, but from a voxel-based analysis. The number of ROIs with $p_{FDR} \leq 0.0025$ were 2, 3, 7, 4, 3, 1, 0, 0, and 11 for the 10-20%, …, 80-90%, and 10-90% cortical depth bins, respectively, and 0 for the voxel-based analysis. The light blue circles denote participants #15-16 from group 2 (ranked based on (**a**)), who had cognitive decline and MCI diagnosis at their year-2 follow-up clinical exam.

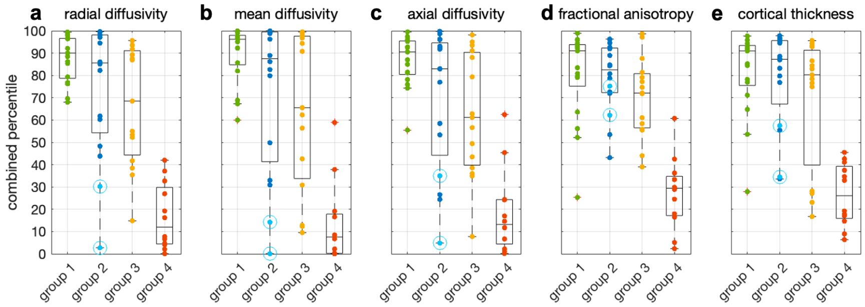

Supplementary Fig. S9. Box plots of combined percentiles averaged over the ROI/depth bin combinations with the most significantly higher dMRI metric for group 4 compared to group 1 ($p_{FDR} \leq 0.0025$) derived from the: (**a**) radial diffusivity (i.e., same as Fig. 3c), (**b**) mean diffusivity, (**c**) axial diffusivity, and (**d**) fractional anisotropy. (**e**) Box plots of combined percentiles averaged over the ROIs with the most significantly smaller cortical thickness for group 4 compared to group 1 ($p_{FDR} \leq 0.0025$). The number of ROI/depth bin combinations (or ROIs) with $p_{FDR} \leq 0.0025$ were 20, 8, 11, 18, and 30, respectively. The light blue circles denote participants #15-16 from group 2 (ranked based on (**a**)), who had cognitive decline and MCI diagnosis at their year-2 follow-up clinical exam.

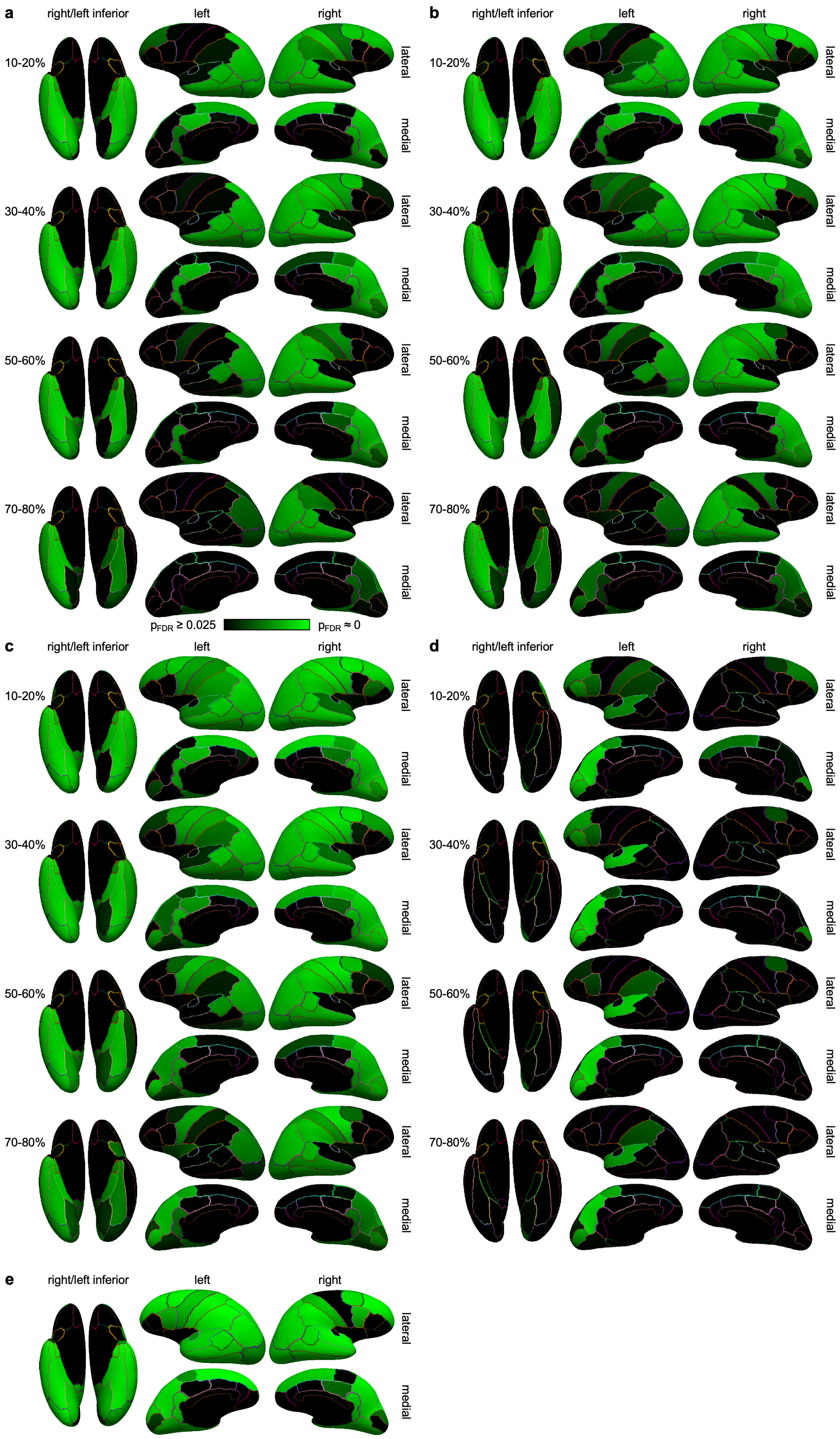

a
right/left inferior
left
right
10-20%
30-40%
50-60%
70-80%
lateral
medial
b
c
d
e
$p_{FDR} \geq 0.025$
$p_{FDR} \approx 0$

Supplementary Fig. S10. Results of the group analysis based on the: (**a**) radial diffusivity (i.e., same as Fig. 2a), (**b**) mean diffusivity, (**c**) axial diffusivity, (**d**) fractional anisotropy, and (**e**) cortical thickness. Inferior, lateral, and medial views of an inflated cortical surface showing the ROIs and cortical depth bins with a significantly higher dMRI metric (or ROIs with a significantly smaller cortical thickness) for group 4 compared to group 1 ($p_{FDR} \leq 0.025$). The number of ROI/depth bin combinations (or ROIs) with $p_{FDR} \leq 0.025$ were 238, 278, 333, 80, and 48, respectively. There were no ROI/depth bin combinations with a significantly lower dMRI metric (or ROIs with a significantly larger cortical thickness) for group 4 compared to group 1.

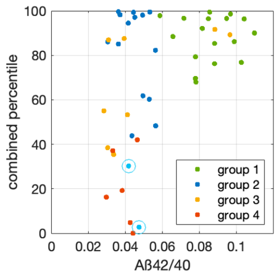

Supplementary Fig. S11. Combined percentiles (derived from the radial diffusivity) averaged over the ROI/depth bin combinations with the most significantly higher radial diffusivity for group 4 compared to group 1 ($p_{FDR} \leq 0.0025$) as a function of the Aß42/40 ratio. The R^2 values were 0.001, 0.055, 0.374, and 0.011 for groups 1–4, respectively, and 0.207 for all groups combined. The light blue circles denote participants #15-16 from group 2, who had cognitive decline and MCI diagnosis at their year-2 follow-up clinical exam.
